# Does an experimentally induced self-association elicit affective self-prioritisation?

**DOI:** 10.1177/17470218221124928

**Published:** 2022-10-14

**Authors:** Gabriela Orellana-Corrales, Christina Matschke, Sarah Schäfer, Ann-Katrin Wesslein

**Affiliations:** 1 Eberhard Karls Universität Tübingen, Tubingen, Germany; 2 Leibniz-Institut für Wissensmedien, Tübingen, Germany; 3 Universität Trier, Trier, Germany

**Keywords:** Self-relevance, bias, IAT, prioritisation

## Abstract

Self-relevant stimuli such as one’s name and face have been demonstrated to influence information processing in both the cognitive and affective domains. It has been observed that recently self-associated stimuli can also influence cognition, but their impact on affect has not been tested yet. In the current study (*N* = 107), we test whether recently self-associated stimuli yield an affective bias and compare the size of the effect with that of familiar self-associated stimuli. A Recoding-Free Implicit Association Test (IAT-RF) presenting self-associated, neutral object-associated, positive, and negative stimuli was used with two groups: one which categorised familiar words as self- and neutral object-associated stimuli, and a second which categorised recently self- and neutral object-associated geometric shapes. In both cases, response times were faster for congruent trials, which mapped response keys as “positive/self” and “negative/neutral object,” than for incongruent trials which mapped response keys as “positive/neutral object” and “negative/self.” The size of the effect yielded by familiar and new self-associated stimuli did not differ. This indicates that experimentally induced self-association can immediately yield an affective bias in favour of the self-associated stimulus.

Our constantly changing environment challenges the cognitive system’s limited capacity to process information. To manage the high quantities of information it constantly receives, the cognitive system filters and prioritises which pieces of information it shall process further. One important characteristic of such prioritised information is the degree to which an item relates to us. That is, stimuli which are associated with the self—such as one’s own name and face—can enhance perception, attention, and memory in comparison to similar stimuli which are associated with someone else (Alexopoulos et al., 2012; Bortolon & Raffard, 2018; Brédart, 2016; Cunningham & Turk, 2017; Yin et al., 2019). It has even been found that self-associated stimuli can affect cognitive information processing when it has only recently been associated with the self; having not yet become an item that is familiarly associated with the self (Schäfer et al., 2015; Schäfer, Wesslein, et al., 2016; Sui et al., 2012). Beyond the cognitive domain, it has also been shown that familiar self-associated stimuli can positively influence attitudes in comparison to equivalent stimuli that are associated with others (Brewer, 1979; Greenwald & Farnham, 2000; Koole et al., 2001; Shu & Peck, 2011). However, this effect on the affective domain has not yet been tested using recently self-associated stimuli. The purpose of this study is to investigate whether this effect generalises to affective measures.

A vast body of research supports the notion that self-associated stimuli are prioritised in cognitive information processing (Bargh, 1982; Constable et al., 2018; Schmitz et al., 2004; Sui et al., 2009; Wójcik et al., 2018). For example, it is a well-known finding that one’s own name is detected with higher probability than simple, neutral words during an auditory shadowing task requiring dichotic listening (Moray, 1959). Similar effects have been demonstrated in other established paradigms such as attentional blindness tasks and visual search (Giesbrecht et al., 2009; Yang et al., 2013). Yet, demonstrations of self-prioritisation in cognitive information processing mostly inherit a confound: the self-relevant stimuli are typically more familiar in comparison to any kind of control stimuli. For example, the favourable detection of one’s own name in an auditory shadowing task (Moray, 1959) may be attributable to the name being much more familiar than other words.

## Experimentally induced self-association causes cognitive self-prioritisation

It has recently been demonstrated that a long-standing history between the self and a previously arbitrary stimulus (e.g., a geometric shape) is not necessary for the latter to elicit cognitive self-prioritisation. To enable the investigation of self-prioritisation in absence of the influence of familiarity, Sui et al. (2012) suggested experimentally inducing the association between the self and a neutral stimulus. Within this experimental paradigm, participants first associated arbitrary geometric shapes to (social) instances that vary in familiarity/neutrality. For example, participants are asked to remember the following: “[the participant’s best friend’s name] is a square; you are a circle; and a stranger is represented by a triangle” (Sui et al., 2012). Having learned this association, participants performed a matching task consisting of the presentation of shape–label pairs. Participants are asked to identify as quickly as possible whether or not each pair is correct in regard to the learned association (i.e., matching; e.g., the label “You” presented with the picture of a circle, or nonmatching; e.g., the label “You” presented with the picture of a square). It has been found that the verification of matching self-associated pairs is faster and more accurate than that of matching other-associated pairs. That is, even the experimental instruction to associate specific geometric shapes to the self was demonstrated to elicit a self-prioritisation effect (SPE) regarding the self-associated shape as opposed to other-associated shapes (Sui et al., 2009, 2012). This finding is interpreted to reflect self-prioritisation in cognitive information processing; hence, in the following, we refer to this effect as “cognitive SPE.” The replication of this effect in various settings has highlighted the flexibility of the self-concept, and shows how spontaneous extensions through self-association can immediately and fundamentally impact cognitive information processing.

Beyond many replications with visual stimuli (Golubickis et al., 2017; Schäfer et al., 2015; Sui & Humphreys, 2015, 2017a) the cognitive SPE has been generalised to simple and previously arbitrary auditory and tactile stimuli (Schäfer, Wesslein, et al., 2016), as well as movements (Frings & Wentura, 2014). Moreover, the cognitive SPE has been shown to be highly robust, prevailing even with poor image quality (Sui et al., 2012) and surviving extinction (Sui & Humphreys, 2017b). Note that the cognitive SPE was initially considered to represent a perceptual effect (Sui et al., 2012) but has now been related to different stages of cognitive information processing, including perception, attention, memory, and decision making (Falbén et al., 2020; Humphreys & Sui, 2015; Macrae et al., 2017; Reuther & Chakravarthi, 2017; Schäfer et al., 2020; Siebold et al., 2015; Stein et al., 2016; Sui & Humphreys, 2017c; Wade & Vickery, 2018).

## Self-prioritisation beyond cognition: the role of self-relevance for attitudes

Until now, effects of experimentally induced self-association beyond the cognitive dimension remain largely unexplored. However, it is well known that self-relevance and self-association strongly impact our feelings and evaluations. According to Boninger and colleagues (1995), self-relevance is a basic characteristic that defines an individual’s attitudes and their subjective importance (see also Howe & Krosnick, 2017; Visser et al., 2016). It is therefore unsurprising that many of our attitudes are influenced by a bias favouring ourselves and self-associated elements over other-associated or neutral persons, institutions, or objects (Brewer, 1979; Brown, 1986; Cunningham & Turk, 2017; Greenwald & Farnham, 2000; Tajfel, 1970; Tajfel et al., 1971). Self-association affects the evaluation of elements with regard to their valence (Gawronski et al., 2007): the self is usually evaluated positively (Krueger, 1998) and—by proxy—self-associated elements tend to be evaluated more positively than neutral elements (Shu & Peck, 2011). Thus, self-association not only impacts cognitive information processing, but also a variety of affective measures.

So far, studies testing valence in the context of newly established self-association have focused on how using self- and other-associated stimuli of positive and negative valence can influence measures of the cognitive SPE. For example, participants have been presented with desirable and undesirable posters which were presented as either self-owned or owned by a friend (Golubickis et al., 2021), they have been asked to associate shapes with good-self/other and bad self/other (Hu et al., 2020), and to associate positive and negative stimuli with themselves and others (Constable et al., 2021) before performing the matching task used to measure the SPE. The results from these studies show that the SPE is enhanced when the self-label is paired with a self-stimulus of positive valence. This has been interpreted as evidence that the SPE activates a self-concept that is positively valent, reflecting a self-enhancement bias (Hu et al., 2020).

However, the valence of newly self- and other-associated stimuli has not yet been directly evaluated using measures of valence, such as the Implicit Association Test (IAT). That is, to the best of our knowledge, no study has yet disentangled the cognitive and affective dimensions of self-prioritisation in recently self-associated stimuli and tested whether arbitrary stimuli induce *affective* self-prioritisation. To close this gap, we will extend the matching task introduced by Sui et al. (2012) to test whether the experimentally induced association of a previously neutral stimulus to the self versus an other causes an affective SPE. Exploring whether the cognitive SPE generalises to affective measures will contribute to a better understanding of the flexibility of the self-concept, since we will learn about how the recent integration of a formerly neutral stimulus causes prioritisation at a general level—including cognitive and affective measures.

Based on the evidence that recently self-associated (more so than other-associated) stimuli are able to induce a cognitive SPE, and on the evidence that familiar self-associated stimuli (more so than other-associated) stimuli induce an affective bias, we predict that both recently and familiar self-associated stimuli will cause an affective SPE (Hypothesis 1). Recent research that has disentangled the cognitive SPE for familiar versus recently associated stimuli has, however, found that the effects of self-association are stronger for stimuli that are familiar compared with recently self-associated stimuli. In a dot-probe task, in which stimuli compete for attention, Orellana-Corrales and colleagues (2020) found that performance advantages for targets that followed self-associated versus stranger-associated stimuli were only observed when familiar stimuli were present. In another line of studies, this finding was also replicated for familiar and recently associated word-stimuli (Orellana-Corrales et al., 2021). Thus, recent evidence suggests that, at least for the cognitive SPE, familiar stimuli are still more effective to elicit biases than recently associated stimuli. Furthermore, the evidence supporting the prioritisation of familiar self-associated stimuli, in comparison to other-associated stimuli, illustrates a robust effect which is present across multiple stages of information processing (e.g., Alexopoulos et al., 2012; Arnell et al., 1999; Bargh, 1982; Moray, 1959; Schmitz et al., 2004; Yang et al., 2013). Such findings contrast with the body of mixed evidence supporting the prioritisation of recently self-associated stimuli in comparison to other-associated stimuli (e.g., Caughey et al., 2021; Dalmaso et al., 2019; Reuther & Chakravarthi, 2017; Schäfer et al., 2017; Siebold et al., 2015; Stein et al., 2016; Wade & Vickery, 2018). We therefore predict that the affective SPE (i.e., a more positive judgement of self- vs. other-associated stimuli) is stronger for familiar stimuli than for recently associated stimuli (Hypothesis 2).

When investigating attitudes and subjective evaluations, it must generally be considered that explicit measures may be distorted by participants’ attempts to hide their real rating of the target subject or by other motivations (Fazio & Olson, 2003). Hence, experimental paradigms that indirectly investigate subjective attitudes and judgements have been developed to avoid these distortions. The IAT (Greenwald et al., 1998) is one tool that indirectly measures attitudes or automatic affect towards specific stimuli. It has become well known for its use to enable, e.g., the measurement of racial biases; where explicit measures typically fail due to social desirability (Hofmann et al., 2005). Within the IAT, participants are simultaneously confronted with two categorisation tasks: one of these tasks includes an affective dimension, requiring participants to classify stimuli as positive or negative (valence categorisation task); the other task comprises the category of interest (target categorisation task). For example, when attempting to measure racial biases, pictures of Black faces and White faces are presented one at a time and participants are asked to classify each face as White or Black. Trials of the valence and target categorisation tasks are mixed, and response categories of both tasks are mapped onto overlapping response keys, with the mapping of keys varying between conditions. In detail, in half of the trials, one target category (e.g., White) is mapped onto the same response key as the words with positive valence, whereas the other target category (e.g., Black) is mapped onto the same response as the words with negative valence. As a result, the mapping is congruent with racial bias towards White people in one condition (i.e., when the categories “White”/ “positive” and “Black”/”negative” are mapped onto the same response key). In the other half of the trials, the target category and valence keys are combined the other way around; a combination which opposes racial bias towards White people (i.e., when the categories “White”/“negative” and “Black”/“positive” are mapped onto the same response key). Faster responses in the former compared with the latter mapping condition are interpreted as racial bias towards White people, because this pattern of reaction times (RTs) suggests an association between race and valence that facilitates responses in the former condition, while hampering responses in the latter condition. The IAT can thus be used to assess the implicit association of automatic affect regarding any category. Generally, analysing categorisation performance as a function of the mapping of response keys enables us to measure the associations with which an individual has been exposed to in their environment, even if they do not explicitly admit to holding such an association (Karpinski & Hilton, 2001).

Using an IAT to measure implicit attitudes towards the self, Greenwald and Farnham (2000) demonstrated that participants’ categorisation responses were faster and more accurate when the response key for familiar self-associated words was mapped to the same response key as pleasant words, and the response key for other-associated words was mapped to the same response key as unpleasant words—reflecting an affective bias towards familiar self-related words. In the current study, we will use the Recoding-Free IAT (IAT-RF; Rothermund et al., 2009) to assess the attitudes towards self-relevant versus other-relevant geometric shapes (see LeBarr & Shedden, 2017, for the measurement of the association of specific objects with the self using a similar procedure). To this end, we will induce self-association of geometric shapes using Sui et al.’s (2012) manipulation and present the shapes in an IAT-RF. Thus, we will be able to determine whether the experimental association of a formerly neutral, experimentally self-associated geometric shape elicits affective prioritisation.

## Overview

The aim of the current study is to investigate whether the experimental instruction to associate neutral shapes with the self versus other instances results in the positive evaluation of the self-associated compared with the other-associated shape. We expect to find an affective component of self-prioritisation. We therefore predict that, in the IAT-RF, the categorisation of stimuli is faster when the mapping of response keys is congruent (combining positive/self and negative/other) compared with an incongruent mapping (positive/other and negative/self combinations). Furthermore, the study aims to compare the size of the affective SPE yielded by recently associated shapes versus familiar words. We expect compatibility effects in the IAT-RF to be greater for familiar words than for recently associated shapes. Finally, the study included the matching task to test whether the established cognitive SPE is replicated in the data.

## Method

The study was approved by the local ethics committee of the Leibniz-Institut für Wissensmedien and preregistered at Open Science Framework (OSF; https://osf.io/v8r2p/).

### Participants

A priori power calculations were made using G*Power (Faul et al., 2007) to establish a minimum sample size for statistically significant results. Previous studies using familiar self-associated and other-associated words have reported large compatibility effects for self/positive and other/negative mappings (Cohen’s *d* = 1.46 in Greenwald & Farnham, 2000 and *d* = 1.11 in Karpinski, 2004). The SPE has also been reported as large in effect size (*dz* > 0.81 in Sui et al., 2012 and *dz* ⩾ 0.58 in Schäfer, Wesslein, et al., 2016). We planned conservatively with a slightly smaller (i.e., medium to large) effect size of  *f* = .30 (Cohen, 1988) for the expected compatibility effect in a priori power analysis. For a one-way analysis of variance (ANOVA) of compatibility effects for two groups (representation type: familiar vs. new), an expected effect size of ƒ = .30, α = .05, a minimum sample size of *N* = 102 is needed to detect an effect with a power of 1 − β = .85.

To allow for dropouts and possible technical issues in online data collection, a total of 136 participants (69 male, *M*_age_ = 28, *SD*_age_ = 8) completed the study. They were randomly assigned to complete one of two versions of the study differing in representation type: familiar (*n* = 71) and new (*n* = 65). Participants were recruited via Prolific, with the criteria of being 18 years of age or older, and native German speakers. All participants had normal or corrected-to-normal vision and were able to complete the study in German. Data from 29 participants were excluded due to the average of their RTs falling within Tukey’s (1977) definition of an outlier when their mean error rates and RTs were compared with the sample distribution of the mean error rates and RTs.

### Design

The study comprised an independent measures design with the between-subjects factor representation type (familiar vs. new) and the within-subjects factor association (self-associated vs. furniture-associated). The dependent variable was the compatibility effect, which was calculated as the difference between incongruent and congruent trials.

### Apparatus and materials

The study was programmed in PsychoPy (Peirce et al., 2019) and run online. Stimuli consisted of visual geometric shapes and words. All stimuli were presented in white against a grey background. All verbal stimuli were presented in Courier New, in 0.06 units of height. Study programming and materials are available on OSF (https://osf.io/v8r2p/).

For the target discrimination task in the IAT-RF, two target categories were used: self-related words and neutral object-related words, for which the category of furniture was chosen. Furniture words (e.g., chair) have been used in previous research as stimuli representing an other (e.g., Klimmt et al., 2010; Schäfer, Frings, & Wentura, 2016). This type of stimuli was chosen because one might argue that the label “other” or “stranger” is less concrete than the label “self” and may therefore fail to elicit the recall of a concrete person. Instead, pieces of furniture refer to specific objects that should be as concrete and easy to recall as the label “self” (see Pinter & Greenwald, 2005 for a discussion on the flexibility and selection of the “other” category in the IAT). The German words “Ich” (I) and the German word “Möbel” [furniture] were used to label these categories. As familiar items to be categorised within these categories, the German words “ich” (I), “mich” (me) and “mir” (me), “mein” (my), and “mich selbst” (myself) represented the category “Ich.” The German words “Tisch” (table), “Bett” (bed), “Stuhl” (chair), “Schrank” (cabinet), and “Sofa” (sofa) were used as familiar items of the category “furniture.” As recently associated items, we used geometric shapes, namely triangles and circles of the same size, filled in five different patterns.

As attribute categories, the German words “positiv” (positive) and “negativ” (negative) were used. The items to be categorised within these categories were the German words “glücklich” [pleasant], “traurig” [sad], “gut” [good], “schlecht” [bad], “schön” [beautiful], hässlich [ugly], Liebe [love], “Hass” [hate], “Lachen” [laughter], “Schmerz” [pain]. Words were selected from the Affective Norms for German Sentiment Terms (Schmidtke et al., 2014) based on their mean valence rating being clearly positive or negative.

### Procedure

At the beginning of the experiment, data were collected about participants’ age, gender, and dominant hand. Participants were then asked to associate one geometric shape (triangle and circle) with each of the categories “self” and “furniture.” The assignment of shapes and categories was balanced across participants. For example, participants were told, “You are a circle, and a piece of furniture is represented by a triangle.” This was done verbally on the screen without any visual presentation of the shapes.

Following, the IAT-RF was presented as described by Rothermund et al. (2009; see Table 1). The task consisted of four blocks which were presented in the same order for all participants, starting with three practice blocks. Participants first performed (a) only the attribute discrimination task (i.e., positive and negative word items had to be categorised as positive vs. negative; 20 trials) with the positive and negative category labels always presented on opposite sides of the top of the screen. The assignment of the attribute dimensions (positive and negative) to the right/left side of the screen was counterbalanced across participants and remained constant throughout the experiment. They then performed (b) only the target discrimination task (i.e., self- and furniture-associated items had to be categorised as self- vs. furniture-associated; 20 trials) with the self/furniture labels always being presented on opposite sides of the top of the screen. Importantly, participants would see either geometric shapes or words relating to the self/furniture during the target discrimination task, depending on the condition they were assigned to. In contrast to the attribute discrimination task, the assignment of the target attributes (self/furniture) to the right/left side of the screen was randomised and counterbalanced across trials. In a final practice block (c), participants then had to work on both tasks simultaneously. Due to the difference regarding the assignment of category labels to the left/right side of the screen in the attribute discrimination task (constant throughout the experiment) and target discrimination task (randomised across trials), the mapping of keys—i.e., whether the self or furniture was associated with the same response key as the positive dimension (e.g., left: self/positive and right: furniture/negative or left: furniture/positive and right: self/negative)—varied between trials. This means that the response keys were not fixed to congruent and incongruent responses, but were rather trial-dependent. Congruent trials were those in which the “Self” and “Positive” category labels were presented together on the same side of the screen (and “Furniture” and “Negative category labels were presented together on the opposite side), and incongruent trials were those in which “Self” and “Negative” category labels were presented together on the same side of the screen (with “Furniture” and “Positive” category labels being presented together on the opposite side). For each item, participants had to perform either the attribute or the target discrimination task, depending on the item at hand (40 trials). Then, the critical block (d) started, in which participants again had to perform both tasks simultaneously (i.e., combined discrimination task; 120 trials).

**Table 1.** Sequence of trial blocks in the self- versus furniture IAT-RF.

Block	Trials	Discrimination task	Categories visible	Category placement	Stimuli categorised
Practice 1	20	Attribute	Positive, negative	Constant	Positive, negative
Practice 2	20	Target	Self, furniture	Randomised across trials	Self, furniture
Practice 3	40	Simultaneous	Positive, negative	Constant	Positive, negative
			Self, furniture	Randomised across trials	Self, furniture
Critical	40	Simultaneous	Positive, negative	Constant	Positive, negative
			Self, furniture	Randomised across trials	Self, furniture

IAT-RF: Recoding-Free Implicit Association Test.

The position of the “positive” and “negative” category labels to the left or right of the screen remained constant across blocks, throughout the whole task. Whether the “positive” or “negative” category was placed to the left or right was randomised and counterbalanced across participants. Stimuli representing “self” and “furniture” were either shapes or words, according to the condition each participant was assigned to.

For each trial, the procedure was the same in all blocks. That is, at the beginning of each block, participants were instructed to place their left index finger on the key “D” and their right index finger on the key “K.” Each trial began with a blank screen (250 ms). The category labels were then presented at the top right and left of the screen (1,250 ms), followed by a fixation cross (500 ms). Next, either an attribute or a target item occurred at the centre of the screen. This item remained on the screen until the participant responded.

After the IAT-RF, participants conducted the matching task that had previously been used to demonstrate the cognitive SPE (e.g., Sui et al., 2012). This task was included to replicate the cognitive SPE. In the matching task, each trial began with a black screen (500 ms) followed by a fixation cross (500 ms). A geometric shape was presented with either the label “self” or “furniture” underneath and remained on the screen until the participant responded, or for a maximum of 1,500 ms. Participants were asked to press “D” if the presented combination was matching according to the instructions provided at the beginning of the experiment, and “K” if the combination was nonmatching. Participants received feedback if their response was incorrect or exceeded 1,500 ms (“incorrect,” “please respond faster”). An initial practice phase presented four trials, and was followed by 140 experimental trials to measure the SPE as established in the literature (Sui et al., 2012). Finally, participants were thanked, debriefed, and received 3.75 lbs for their participation through the online platform.

## Results

The collected data and analysis scripts are available on OSF (https://osf.io/v8r2p/).

### IAT-RF

**Figure 1.**
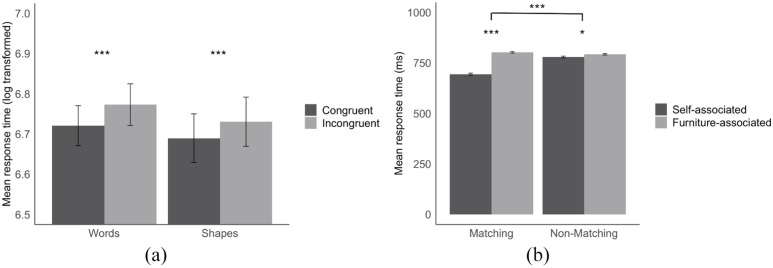
Mean response times in the self/furniture positive/negative IAT-RF (a) as a function of trial type (congruent vs. incongruent) for groups categorising different types of self- and furniture-associated stimuli (words vs. shapes), and mean response times in the matching task (b) as a function of shape (self-associated vs. furniture-associated) and trial type (matching vs. nonmatching). Error bars represent standard errors. ***p < .001. *p < .05.

The IAT-RF data were analysed as described by Rothermund et al. (2009).^1^ First, compatibility effects were calculated for the RT data of each representation type condition (i.e., familiar words and new shapes) in the IAT-RF. This consisted of log-transforming the mean of RTs for incongruent and congruent trials, and then subtracting the result for congruent trials from the result for incongruent trials (see Figure 1). These were then tested by comparing them between the two representation types (familiar words vs. new shapes). In this analysis strategy, the combination of categories that are supposedly congruent (self/positive and furniture/negative) are compared with the combination of categories that are supposedly incongruent (self/negative and furniture/positive). That is, all congruent and incongruent trials are included in the calculation of compatibility effects, even those from the attribute discrimination task in which positive and negative words are categorised. The IAT works under the assumption that if two concepts share a response key, the categorisation task will be much easier (i.e., responses will be faster) if the concepts are strongly associated than if they are weakly associated (Greenwald et al., 2002). Thus, the comparison between congruent and incongruent trials is done to measure the strength of the association between both pairs of categories.^2^ This means that the association is evaluated in both directions, with “self” being associated with “positive,” and “positive” being associated with “self.”

Importantly, due to the use of complementary pairs of categories in the IAT, its results can only be interpreted as relative strengths of associations in comparison to one another (see Greenwald & Farnham, 2000; Karpinski & Steinman, 2006). This means that there is no “absolute zero”; rather than observing that “self” is associated with “positive,” and “furniture” is associated with “negative,” what the congruency effect indicates is that “self’ is more strongly associated with “positive” in comparison to “furniture,” and that “self” is less strongly associated with “negative” in comparison to “furniture.”

#### Compatibility effects

As expected in Hypothesis 1, a significant compatibility effect was found in the condition in which self/furniture were represented by familiar word-stimuli, *t*(54) = 5.69, *p* < .001, *dz* = 0.75. That is, responses were faster when the response key mapping was congruent (*M* = 6.72, *SD* = .18)—in which positive/self word labels were mapped to one response key and negative/furniture word labels were mapped to the other key—than when the mapping was incongruent (*M* = 6.77, *SD* = .19), with positive/furniture word labels being mapped to one key, and negative/self word labels being mapped to another key. Again in line with Hypothesis 1, for the recently associated stimuli, a significant compatibility effect was observed, *t*(51) = 5.18, *p* < .001, *dz* = 0.70, with RTs being faster when the mapping was congruent (*M* = 6.69, *SD* = .22), than when the mapping was incongruent (*M* = 6.73, *SD* = .22). That is, responses were facilitated when the two response keys were mapped as positive/self-associated shapes and negative/furniture-associated shapes than when the two response keys were mapped as positive/furniture-associated shapes and negative/self-associated shapes.

To test whether the effect differs between the familiar (i.e., words) and the recently associated (i.e., shapes) stimuli (as expected in Hypothesis 2), the compatibility effect data were submitted to a one-way ANOVA with two groups (word vs. shape). Other than expected, the analysis did not reveal a difference in compatibility effects between word and shape stimuli, *F*(1, 105) = 0.84, $\eta_{p}^{2}$ = .008, *p* = .360, reflecting that familiar and recently associated stimuli were comparable in their facilitation of responses for congruent versus incongruent mapping. A follow-up Bayesian analysis using JASP (JASP Team, 2020) provided moderate evidence for the null hypothesis that compatibility effects did not differ between word and shape stimuli, BF_01_ = 3.35.

Taken together, these IAT-RF results indicate that self-associated stimuli are evaluated more positively than stimuli that are associated with neutral instances. This finding holds when self- versus other-related familiar stimuli are compared, but is also comparable, when self- versus other-related stimuli were only just recently associated.

### Matching task

The analysis of the matching task followed the established analyses in the literature (e.g., Sui et al., 2012): first, the average RTs were analysed, second, a sensitivity analysis was conducted.

#### Average RTs

A 2 (shape: self-associated vs. furniture-associated) × 2 (trial type: matching vs. nonmatching) within-participants ANOVA was carried out to analyse the RT data (see Figure 1). Both main effects of shape, *F*(1, 106) = 199.28, *p* < .001, $\eta_{p}^{2}$ = .65, and trial type, *F*(1, 106) = 77.08, *p* < .001, $\eta_{p}^{2}$ = .42, were statistically significant. The shape × trial type interaction, *F*(1, 106) = 111.13, *p* < .001, $\eta_{p}^{2}$ = .51, was significant as well.

RTs from matching and nonmatching trials were separately submitted to a single-factor (shape: self-associated vs. furniture-associated) within-participants ANOVA to follow-up on the interaction effect. The analysis on matching trials revealed a significant main effect, *F*(1, 106) = 255.76, *p* < .001, $\eta_{p}^{2}$ *=* .71, reflecting a significant SPE. In detail, RTs were faster when responding to matching self-associated shape–label pairs (*M* = 694, *SD* = 91) than to matching furniture-associated shape–label pairs (*M* = 802, *SD* = 103). The analysis on nonmatching trials also revealed a significant main effect, *F*(1, 106) = 5.90, *p* = .017, $\eta_{p}^{2}$ *=* .05. That is, RTs were faster when responding to nonmatching pairs of the self-associated shape and furniture-associated label (*M* = 779, *SD* = 106) than to nonmatching pairs of the furniture-associated shape and self-associated label (*M* = 793, *SD* = 100). The significant interaction effect can thus be attributed to self-associated shapes (compared with furniture-associated shapes) enhancing RTs to a greater degree in matching trials (compared with nonmatching trials). We did not observe a correlation between SPE measures and IAT-RF measures, *r* = .04, *p* = .718.

#### Sensitivity measure *d*ʹ

Error rates were analysed by using signal detection sensitivity measures for each shape. To calculate these measures, responses in matching trials were categorised as hits when correct, and as misses when incorrect; responses in nonmatching trials were categorised as correct rejections when correct, and as false alarms when incorrect. Following the loglinear approach to account for cases with 100% hits or 0% false alarms, 0.5 was added to the number of hits and the number of false alarms, and 1 was added to the number of signal trials and the number of noise trials prior to calculating the rates for hits and false alarms. We followingly calculated the measure of sensitivity *dʹ* and submitted it to a single-factor (shape: self-associated vs. furniture-associated) ANOVA. The analysis revealed a significant effect, *F*(1, 106) = 4.63, *p* = .034, $\eta_{p}^{2}$ = .04, indicating higher sensitivity towards self-associated shapes (*M* = 2.34, *SD* = 1.04) than furniture-associated shapes (*M* = 2.19, *SD* = 1.08). Taken together, these analyses of the matching task reveal a cognitive SPE in line with the established evidence: recently self-associated stimuli received an attentional prioritisation compared with recently other-associated stimuli.

## Discussion

The aim of this study was to test whether recently self-associated stimuli would elicit an affective SPE. More specifically, we investigated whether the experimental instruction to associate neutral shapes with the self and other instances resulted in the positive evaluation of the self-associated shape compared with the other-associated shape. Furthermore, we aimed to compare the size of the affective SPE between recently self-associated stimuli (i.e., shapes) and established, familiar self-stimuli (i.e., words).

As expected, when self- and furniture-associated shapes were used in the IAT-RF, the categorisation of stimuli was faster in congruent trials, where response keys were mapped as positive/self and negative/furniture, than in incongruent trials where response keys were mapped as positive/furniture and negative/self. The results indicate that newly acquired self-associations are enough to cause an associative difference—reflecting a positive affective bias towards newly self-associated stimuli. Interestingly (and contrary to what was expected), the size of the affective SPE was as strong for newly self-associated stimuli (i.e., shapes) as for familiar self-associated stimuli (i.e., words).

### Affective self-prioritisation

The present data demonstrate that even recently self-associated stimuli yield an affective prioritisation. The finding replicates and extends prior research which demonstrated a self-prioritisation bias in affective measures. A large body of research demonstrates that self-relevance is a strong guide for attitudes and valence judgements (e.g., Brewer, 1979; Brown, 1986; Cunningham & Turk, 2017; Gawronski et al., 2007; Tajfel, 1970; Tajfel et al., 1971). Using indirect measures, Greenwald and Farnham’s (2000) finding in the IAT demonstrated an affective bias towards the self when familiar stimuli were used. The present data replicates this affective SPE for familiar words but, more importantly, it demonstrates that even self-related stimuli without prior history of representing the self can elicit an affective SPE. The use of geometric shapes that were just recently associated with the self and a neutral other (i.e., furniture) extends former results by demonstrating for the first time that this affective bias towards the self is not limited to stimuli that are familiarly associated with the self. Rather, the tendency to favour self-related stimuli seems a robust effect that can be elicited by a simple instruction to associate a formerly neutral shape to the self. As previously mentioned, the use of complementary pairs in the IAT implies that results can only be interpreted as relative strengths of associations—an important consideration to keep in mind when discussing the results. That is, what we observe in our results is simply that “self’ is *more strongly* associated with “positive” in comparison to “furniture,” and that “self’’ is *less strongly* associated with “negative” in comparison to “furniture.” In this case, the pattern of results reflecting faster RTs for congruent than incongruent furniture-trials most likely reflects a self-response bias, as has been previously observed in categorisation tasks (e.g., Golubickis et al., 2018) or a self-enhancing bias as reflected in categorisation tasks using valence self-stimuli (e.g., Hu et al., 2020) rather than an existing association of “furniture” as “negative.”

The finding that formerly neutral symbols are tagged once they are associated with the self and enriched with positive value is comparable with previous research that used the minimal group paradigm. In this paradigm, the allocation to a neutral, formerly unknown group enhances affective biases between groups (e.g., Brewer, 1979; Tajfel, 1970; Tajfel et al., 1971). The present approach is even more parsimonious. Whereas the allocation to an ostensible group might facilitate and strengthen self-association because it triggers concepts such as belonging, collaboration, and competition, the association of the self to a geometric figure (especially as the figures were counterbalanced) is unlikely to trigger any special meaning or prior belonging experiences. Thus, the present study tests for the first time whether previously neutral and arbitrary stimuli which are associated with the self can immediately induce an affective bias.

### Familiar versus recently associated stimuli

Contrary to what was expected, we did not observe a significant difference between the size of the effect yielded by familiar self-associated words and the size of the effect yielded by recently self-associated shapes. That is, we observed that familiar self-associated words and recently self-associated shapes yielded effects of a similar degree in enhancing implicit attitudes towards the self. Thus, our study provides preliminary evidence that familiar and new representations of the self can be equally effective at inducing an affective bias in an IAT. This finding is not fully in line with previous research on the cognitive SPE. Recent research that disentangled familiar labels and recently associated shapes demonstrated that the cognitive SPE can only be found when familiar stimuli are present (Orellana-Corrales et al., 2020). This pattern was found for both recently associated shapes and for recently associated letter-combinations (Orellana-Corrales et al., 2021). In the present data, however, both familiar and recently associated self-stimuli elicited affective prioritisation. There are methodological differences that may have some influence in the discrepancy of the results. First, representation type was manipulated between participants rather than within. As suggested by Orellana-Corrales and colleagues (2020), the simultaneous presentation of new and familiar self-associated stimuli may hinder the potential of new self-associated stimuli to impact cognition. Thus, it may be possible that we observed similar effects for familiar and new representations in affect because each participant categorised only one representation type. One other possible explanation for this discrepancy can be due to the difference in how these mechanisms function. For example, cognitive tasks—particularly those focused on earlier stages of information processing such as perception and attention—measure a certain physical automaticity that is usually strengthened by repetition (see Reuther & Chakravarthi, 2017). On the contrary, affective measures can tap into associations that work at a conceptual level, where the association can endow one entity with another’s specific characteristics which are already known. Thus, the equivalence between two entities can be established quickly without requiring as much repetition. As research exploring the influence of stimuli valence on the SPE has demonstrated that activations of the self-concept are usually positively valenced (possibly due to a self-enhancement bias; Constable et al., 2021; Golubickis et al., 2019; Hu et al., 2020) it seems reasonable that a new self-associated stimuli can quickly yield positive affect. Considering this, it seems worthwhile to broaden the study of SPEs of recently associated stimuli by using various measures. Although the IAT-RF in the present study was deliberately chosen to indirectly assess affective bias, future studies should test whether the findings replicate with direct measures. In addition, it seems a promising approach to assess behavioural self-prioritisation of recently self-associated stimuli to understand whether the self-association translates into behaviour, and to test how long such effects would hold. All in all, the comparison of the SPE between familiar versus new representations of the self using different measures that tap into cognitive, affective, and behavioural consequences of self-associations seems a future avenue worth exploring.

Due to the indirect nature of the measures that were used, one might criticise that participants did not associate the shape with the self, but with the word “self.” In other words, the manipulation and measurement might capture an association with the associated self-word. Due to the fact that the IAT and the matching task work with self-symbols (i.e., words that represent the self), it is not possible to say with certainty whether we are observing a “true” association of a new stimulus to the self, or an “association to an association.” In fact, it could be considered that the observed effects are due to recoding or working memory, which is one of the strongly backed suggestions for the cognitive underpinning of the cognitive SPE (Falbén et al., 2020; Siebold et al., 2015; Wade et al., 2018). In our study, we did not observe a correlation between SPE and congruency effects—suggesting that congruency effects are independent from the cognitive underpinnings of the SPE. However, further research is needed to define this. An interesting direction for future studies would be to build on the literature using functional magnetic resonance imaging (fMRI) in the context of self- versus other-associated stimuli (e.g., Chen & Huang, 2017; Murray et al., 2012, 2015; Sui et al., 2015) to compare the neural response to highly familiar self-associated stimuli such as one’s own face, with that of experimentally induced self-associated stimuli.

### Strengths and limitations

The present study has several strengths and limitations. One strength is that it is the first to test the potential of recently associated stimuli for affective self-prioritisation. At the same time, it replicates and compares the affective self-prioritisation of familiar stimuli with the recently associated stimuli. Using the IAT-RF makes use of an indirect measure that is less prone to impression management or demand characteristics (e.g., Fazio & Olson, 2003). In addition, the inclusion of the matching task and the replication of the usual pattern in this task (Schäfer et al., 2015; Schäfer, Wesslein, et al., 2016; Sui et al., 2012) provide further evidence that inducing self-association leads to cognitive prioritisation in a matching task, and that this effect is highly robust. This finding also allowed us to confirm that the manipulation used in our study was effective and comparable to former research.

One limitation is, of course, the use of only one affective measure and the choice of the IAT itself. Although the IAT is an established measure to capture attitudes indirectly, it has also been widely criticised (see, e.g., De Houwer et al., 2009; Fiedler et al., 2006 for discussion). Another limitation is the selection of representative stimuli for the categories “self” and “other.” Considering the multiple ways in which “self” can be represented, it becomes clear that the familiarity of symbols can have various degrees, ranging from new and unfamiliar (e.g., shapes, new letter-strings, avatars, or nicknames) to highly familiar (e.g., one’s face, names). The stimuli that were used as familiar symbols in the present study (i.e., pronouns representing the self) may be familiar, but might not range among the top familiar symbols that represent the self. Therefore, future research should replicate the present finding with the use of other familiar and new stimuli. In addition to selecting appropriate “self” and “other” categories, selecting stimuli that can both represent each category and be matched across categories to be fairly equivalent is challenging. In our study, intra-class similarity in the word condition was not equivalent. Finally, the study presents what can only be considered as preliminary evidence of this effect in an experimental setting. Further research that replicates and builds on this study is required to learn more about the dynamics of this effect in applied contexts.

## Conclusion

In summary, our study presents novel evidence that even recently self-associated stimuli are prioritised affectively in comparison to other-associated stimuli, just like familiar self-associated stimuli are evaluated more positively than other-associated stimuli. Thus, the cognitive SPE which had been found in prior research for recently self-associated stimuli was extended to the affective dimension by showing that stimuli are immediately enriched with positive valence once they are associated with the self. This finding demonstrates, once again, that self-relevance is a powerful characteristic that guides information processing as well as spontaneous affective responses and attitudes towards stimuli.

## Supplemental Material

sj-docx-1-qjp-10.1177_17470218221124928 – Supplemental material for Does an experimentally induced self-association elicit affective self-prioritisation? Click here for additional data file. Supplemental material, sj-docx-1-qjp-10.1177_17470218221124928 for Does an experimentally induced self-association elicit affective self-prioritisation? by Gabriela Orellana-Corrales, Christina Matschke, Sarah Schäfer and Ann-Katrin Wesslein in Quarterly Journal of Experimental Psychology

## Notes

1 For deviations from the preregistered analysis, see the online Supplementary Material “Transparent Changes.”

2 Instead of using the difference between congruent and incongruent trials as a dependent measure in *t* tests, it is also possible to analyse the performance in the target discrimination task of the Recoding-Free Implicit Association Test (IAT-RF) with 2 (association: self vs. furniture) × 2 (trial type: congruent vs. incongruent) × 2 (representation type: familiar vs. new) analyses of variances (ANOVAs). For ease of readability and conciseness, we chose to present the analysis as suggested by Rothermund et al. (2009) and have reported the alternative analysis using ANOVAs in the Supplementary Material.
