# Supplementary material for: Does an experimentally induced self-association elicit affective self-prioritisation?
Source: Q J Exp Psychol (Hove). 2022 Oct 14;76(6):1379–90. doi: 10.1177/17470218221124928 (PMC10196926; doi:10.1177/17470218221124928)
Supplement: sj-docx-1-qjp-10.1177_17470218221124928 – Supplemental material for Does an experimentally induced self-association elicit affective self-prioritisation? [file sj-docx-1-qjp-10.1177_17470218221124928.docx]

Supplementary Material for:

**The Dual Mechanisms of Cognitive Control (DMCC) project:**

**Validation of an on-line behavioral task battery**

Rongxiang Tang^1^, Julie M. Bugg^1^, Jean-Paul Snijder^2^, Andrew R. A. Conway^2^, Todd S. Braver^1*^

^1^Department of Psychological and Brain Sciences, Washington University in St. Louis

^2^Division of Behavioral & Organizational Sciences, Claremont Graduate University

*Corresponding Author:

Todd S. Braver

Email: tbraver@wustl.edu

Telephone: 314-935-5143

Address: Washington University,

Campus Box 1125,

One Brookings Drive,

St. Louis, Missouri 63130-4899

# **Supplemental Table 1. Stroop Results across Trial Types and Conditions**

| Condition | Trial Type | RT Mean (SD) | Error Mean (SD) |
| --- | --- | --- | --- |
| Baseline | Biased | Congruent: 768.51 (372.96)  Incongruent: 920.27 (381.32)  Stroop Effect: 151.76 (69.57) | Congruent: 0.03 (0.06)  Incongruent: 0.07 (0.08)  Stroop Effect: 0.03 (0.05) |
|  | PC-50 | Congruent: 792.41 (380.73)  Incongruent: 910.60 (376.78)  Stroop Effect: 118.19 (74.30) | Congruent: 0.03 (0.06)  Incongruent: 0.05 (0.06)  Stroop Effect: 0.02 (0.04) |
| Proactive | Biased | Congruent: 772.58 (368.51)  Incongruent: 856.32 (365.03)  Stroop Effect: 83.74 (53.43) | Congruent: 0.01 (0.04)  Incongruent: 0.03 (0.04)  Stroop Effect: 0.01 (0.03) |
|  | PC-50 | Congruent: 779.85 (369.01)  Incongruent: 872.81 (369.54)  Stroop Effect: 92.96 (68.66) | Congruent: 0.01 (0.05)  Incongruent: 0.03 (0.05)  Stroop Effect: 0.01 (0.03) |
| Reactive | Biased | Congruent: 761.45 (414.05)  Incongruent: 854.99 (397.29)  MC Filler: 761.80 (381.44)  Stroop Effect: 93.54 (66.24) | Congruent: 0.02 (0.04)  Incongruent: 0.04 (0.05)  MC Filler: 0.00 (0.00)  Stroop Effect: 0.03 (0.03) |
|  | PC-50 | Congruent: 775.38 (386.95)  Incongruent: 902.38 (378.99)  Stroop Effect: 127.01 (73.96) | Congruent: 0.02 (0.05)  Incongruent: 0.04 (0.06)  Stroop Effect: 0.02 (0.03) |
| Condition | Derived Measure | Trial Type | RT Mean (SD) |
| Proactive | Congruency Cost | Biased  PC-50 | 4.07 (171.43)  -12.56 (167.72) |
|  | Transfer Cost | – | 9.22 (58.63) |
| Reactive | Congruency Cost | Biased  PC-50 | -7.06 (155.40)  -17.03 (121.78) |
|  | Transfer Cost | – | 33.48 (47.91) |

# **Supplemental Table 2. AX-CPT Results across Trial Types and Conditions**

| Condition | Trial Type | RT Mean (SD) | Error Mean (SD) |  | |
| --- | --- | --- | --- | --- | --- |
| Baseline | AX  AY  A-nogo  BX  BY  B-nogo | 465.04 (80.45)  548.77 (73.45)  –  314.93 (148.53)  324.66 (66.51)  – | 0.07 (0.10)  0.06 (0.07)  0.13 (0.13)  0.19 (0.18)  0.01 (0.03)  0.21 (0.17) | |  |
| Proactive | AX  AY  A-nogo  BX  BY  B-nogo | 422.73 (86.61)  548.96 (85.40)  –  474.79 (121.82)  418.34 (68.78)  – | 0.05 (0.08)  0.20 (0.19)  0.18 (0.19)  0.10 (0.11)  0.01 (0.02)  0.33 (0.22) | |  |
| Reactive | AX  AY  A-nogo  BX  BY  B-nogo | 449.38 (82.66)  572.11 (88.97)  –  565.90 (117.92)  435.83 (72.53)  – | 0.07 (0.08)  0.07 (0.08)  0.09 (0.09)  0.14 (0.15)  0.01 (0.03)  0.13 (0.12) | |  |
| Condition | Derived Measure | RT Mean (SD) | Error Mean (SD) | | Z-scores Mean (SD) |
| Baseline | A-cue Bias  BX Interference  d’-context | –  91.11 (106.84)  – | –  0.19 (0.17)  – | | 0.03 (0.30)  –  2.61 (0.93) |
|  | PBI | 0.01 (0.08) | -0.35 (0.44) | | – |
| Proactive | A-cue Bias  BX Interference  d’-context | –  56.45 (74.55)  – | –  0.11 (0.10)  – | | 0.42 (0.46)  –  3.12 (0.88) |
|  | PBI | 0.10 (0.01) | 0.18 (0.53) | | – |
| Reactive | A-cue Bias  BX Interference  d’-context | –  130.07 (77.40)  – | –  0.14 (0.14)  – | | 0.06 (0.31)  –  2.84 (0.85) |
|  | PBI | 0.01 (0.07) | -0.19 (0.52) | | – |

# **Supplemental Table 3. Cued Task-Switching Results across Trial Types and Conditions**

| Condition | Trial Type | RT Mean (SD) | Error Mean (SD) |
| --- | --- | --- | --- |
| Baseline | Biased (Non-Incentivized)  Unbiased (Non-Incentivized) | Congruent: 968.16 (257.56)  Incongruent: 1008.66 (267.56)  TRCE: 40.51 (126.90)  Switch Cost: 38.63 (76.68)  Congruent: 1005.86 (301.22)  Incongruent: 1052.15 (254.24)  TRCE: 46.30 (132.36)  Switch Cost: 39.96 (115.66) | Congruent: 0.05 (0.06)  Incongruent: 0.11 (0.13)  TRCE: 0.06 (0.11)  Switch Cost: 0.02 (0.04)  Congruent: 0.07 (0.09)  Incongruent: 0.13 (0.11)  TRCE: 0.06 (0.10)  Switch Cost: 0.03 (0.08) |
| Proactive | Biased (Non-Incentivized)  Unbiased (Incentivized) | Congruent: 746.98 (197.89)  Incongruent: 793.46 (229.47)  TRCE: 46.48 (99.92)  Switch Cost: 33.56 (49.39)  Congruent: 694.74 (208.80)  Incongruent: 723.47 (217.78)  TRCE: 28.73 (63.02)  Switch Cost:12.56 (61.62) | Congruent: 0.05 (0.04)  Incongruent: 0.13 (0.12)  TRCE: 0.09 (0.12)  Switch Cost: 0.01 (0.04)  Congruent: 0.10 (0.11)  Incongruent: 0.23 (0.12)  TRCE: 0.13 (0.13)  Switch Cost: 0.03 (0.09) |
| Reactive | Biased (Non-Incentivized)  Unbiased (Incentivized) | Congruent: 1054.53 (308.78)  Incongruent: 1134.98 (319.83)  TRCE: 80.45 (166.06)  Switch Cost: 58.31 (82.11)  Congruent: 1175.97 (366.90)  Incongruent: 1228.09 (322.15)  TRCE: 52.12 (137.97)  Switch Cost: 37.93 (127.82) | Congruent: 0.02 (0.02)  Incongruent: 0.06 (0.09)  TRCE: 0.04 (0.09)  Switch Cost: 0.01 (0.03)  Congruent: 0.01 (0.03)  Incongruent: 0.07 (0.08)  TRCE: 0.05 (0.08)  Switch Cost: 0.03 (0.07) |

# **Supplemental Table 4. Sternberg Results across Trial Types and Conditions**

| Condition | Trial Type | RT Mean (SD) | Error Mean (SD) |
| --- | --- | --- | --- |
| Baseline | NN | Critical: 839.11 (165.20)  High: 883.58 (165.00) | Critical: 0.04 (0.08)  High: 0.07 (0.08) |
|  | NP | Critical: 890.12 (166.92)  High: 909.63 (170.34) | Critical: 0.14 (0.13)  High: 0.18 (0.11) |
|  | RN | Critical: 967.16 (186.84)  High: 1039.74 (236.12) | Critical: 0.20 (0.16)  High: 0.39 (0.19) |
| Proactive | NN | Critical: 843.25 (170.37)  Low: 826.06 (162.12) | Critical: 0.05 (0.08)  Low: 0.05 (0.07) |
|  | NP | Critical: 858.48 (157.29)  Low: 788.47 (148.90) | Critical: 0.12 (0.11)  Low: 0.05 (0.06) |
|  | RN | Critical: 1037.27 (238.65)  Low: 870.60 (209.79) | Critical: 0.27 (0.22)  Low: 0.07 (0.14) |
| Reactive | NN | Critical: 851.44 (157.70)  High: 922.91 (189.34) | Critical: 0.03 (0.08)  High: 0.06 (0.13) |
|  | NP | Critical: 871.39 (149.05)  High: 902.71 (144.34) | Critical: 0.10 (0.10)  High: 0.19 (0.11) |
|  | RN | Critical: 949.53 (161.04)  High: 1019.81 (164.22) | Critical: 0.13 (0.13)  High: 0.28 (0.16) |
| Condition | Derived Measure | RT Mean (SD) | Error Mean (SD) |
| Baseline | Recent Negative Effect | Critical: 128.05 (110.77)  Non-critical: 174.25 (190.14) | Critical: 0.16 (0.15)  Non-critical: 0.32 (0.18) |
| Proactive |  | Critical: 194.77 (148.99)  Non-critical: 47.27 (138.02) | Critical: 0.22 (0.20)  Non-critical: 0.02 (0.12) |
| Reactive |  | Critical: 98.10 (96.40)  Non-critical: 94.62 (138.55) | Critical: 0.10 (0.12)  Non-critical: 0.22 (0.16) |
